# Non-Replication of Genome-Wide Based Associations between Common Variants in *INSIG2* and *PFKP* and Obesity in Studies of 18,014 Danes

**DOI:** 10.1371/journal.pone.0002872

**Published:** 2008-08-06

**Authors:** Camilla H. Andreasen, Mette S. Mogensen, Knut Borch-Johnsen, Annelli Sandbæk, Torsten Lauritzen, Thorkild I. A. Sørensen, Lars Hansen, Katrine Almind, Torben Jørgensen, Oluf Pedersen, Torben Hansen

**Affiliations:** 1 Steno Diabetes Center, Copenhagen, Denmark; 2 Medical and Science, Developmental Projects, Novo Nordisk A/S, Bagsværd, Denmark; 3 Research Centre for Prevention and Health, Glostrup University Hospital, Glostrup, Denmark; 4 Faculty of Health Science, University of Aarhus, Aarhus, Denmark; 5 Department of General Practice, University of Aarhus, Aarhus, Denmark; 6 Bristol-Myers Squibb Co., Princeton, New Jersey, United States of America; 7 Institute for Preventive Medicine, Copenhagen University Hospitals, Centre for Health and Society, Copenhagen, Denmark; University of Las Palmas de Gran Canaria, Spain

## Abstract

**Background:**

The *INSIG2* rs7566605 and *PFKP* rs6602024 polymorphisms have been identified as obesity gene variants in genome-wide association (GWA) studies. However, replication has been contradictory for both variants. The aims of this study were to validate these obesity-associations through case-control studies and analyses of obesity-related quantitative traits. Moreover, since environmental and genetic factors may modulate the impact of a genetic variant, we wanted to perform such interaction analyses. We focused on physical activity as an environmental risk factor, and on the GWA identified obesity variants in *FTO* (rs9939609) and near *MC4R* (rs17782313) as genetic risk factors.

**Materials and Methods:**

The four variants were genotyped in a combined study sample comprising a total of 18,014 subject ascertained from, the population-based Inter99 cohort (*n* = 6,514), the ADDITION screening cohort (*n* = 8,662), a population-based study sample (*n* = 680) and a type 2 diabetic patient group (*n* = 2,158) from Steno Diabetes Center.

**Results:**

No association with overweight, obesity or obesity-related measures was shown for either the *INSIG2* rs7566605 or the *PFKP* rs6602024 variants. However, an interaction between the *INSIG2* rs7566605 variant and the level of self-reported physical activity (*p*_Int_ = 0.004) was observed. A BMI difference of 0.53 (SE 0.42) kg/m^2^ was found when comparing physically passive homozygous C-allele carriers with physically passive G-allele carriers. No interactions between the two variants and *FTO* rs9939609 and *MC4R* rs17782313 were observed.

**Conclusions:**

The *INSIG2* rs7566605 and *PFKP* rs6602024 polymorphisms play no apparent role in the development of common forms of obesity in the Danish population. However, if replicated, the *INSIG2* rs7566605 may influence the level of BMI in combination with the level of physical activity.

## Introduction

The identification of genetic variants contributing to common forms of obesity has been challenging using traditional strategies for selection of candidate genes. However, within the last few years rapid advancements have made genome-wide association (GWA) studies feasible and these studies have provided new insight into the pathogenesis of a number of complex polygenic disorders. Several GWA studies have been performed on obesity, and have resulted in the identification of new potential obesity susceptibility gene variants. The rs7566605 located 10 kb upstream of the insulin induced gene 2 (*INSIG2*), was the first common obesity-related gene variant to be identified through a GWA approach, including 694 individuals [1]. Approximately 10% of the study population carried the risk CC-genotype, and were on average 1 body mass index (BMI) unit heavier than heterozygous or homozygous G-allele carriers. The initial finding was validated in four out of five replication samples, involving a total of 9,881 individuals, with a combined odds ratio (OR) of 1.22 (1.05–1.42) under a recessive model [1]. Moreover, an independent GWA scan, comprising 1,000 unrelated U.S. Caucasians, has observed an association between the *INSIG2* rs7566605 variant and obesity [2]. The association of *INSIG2* rs7566605 with BMI has further been observed in five out of nine independent large cohorts from eight populations across multiple ethnicities, and a meta-analysis of all nine cohorts, comprising nearly 17,000 individuals also confirmed the association [3]. Several other independent studies have, however, failed to validate the originally proposed association between rs7566605 and obesity when examining a total of 28,043 Caucasian [4]–[9], 2,292 Indian [9], [10] or 6,477 Asian individuals [11]–[13]. INSIG2 is the predominant protein isoform in adipocytes [14], and regulates the transcription of genes promoting fatty acid synthesis and adipogenesis [15], [16], making *INSIG2* a good biological candidate gene for obesity.

The rs6602024 in the platelet type phosphofructokinase (*PFKP*) gene was identified in a GWA scan of obesity-related traits in 4,741 individuals, from a genetically isolated population of Sardinia, to associate strongly with BMI, body weight and hip circumference under an additive model [17]. The association was, however, not validated in independent replication cohorts, including a total of 3,467 subjects, even though homozygous carriers of the minor A-allele on average were ∼1–3 BMI units heavier than homozygous carriers of the G-allele [17].

*PFKP* encodes a phosphofructokinase acting as a key regulatory enzyme in the glycolysis. Enhanced activity of *PFKP* might alter the balance between glycolysis and glycogen production, increasing the glucose utilization and thereby lipogenesis, ultimately leading to obesity [17]–[19].

GWA studies have already proved successful and reported novel, unanticipated genetic variants contributing to disease risk. A now well-established link to obesity includes variants within the first intron of the fat mass and obesity associated gene (*FTO*) [17], [20]–[22]. The consistency of replication across several samples of Caucasian origin [23]–[26] and lately in other ethnic samples [27], [28] has considerably strengthened the important contribution of *FTO* to obesity. *FTO* has upon identification been found to encode a 2-oxoglutarate-dependent nucleic acid demethylase [29], [30], however, the link between this enzyme and the development of obesity remains to be elucidated. We have previously validated the association between the rs9939609 variant within *FTO* and obesity measures, and interestingly, an interaction with physical activity was demonstrated to differentiate the degree of body fat accumulation between genotype groups [23].

A large-scale meta-analysis of GWA data available for 16,876 individuals identified a variant (rs17782313) mapping 188 kb downstream of the gene encoding melanocortin-4 receptor (*MC4R*) to influence on BMI and the finding were replicated in large-scale studies of 77,228 individuals, both adults and children, with a combined OR for obesity of 1.12 (1.08–1.16), *p* = 5.2×10^−9^ [31]. Although mapping hundreds of kilo bases from the coding sequence of *MC4R* the identified variant presumable disrupt the transcriptional control of *MC4R*. MC4R is involved in appetite regulation and represent a compelling biological candidate, as rare coding mutations in the gene are a cause of monogenic obesity in humans [32].

Statistically powered replications of GWA findings are essential to determine the true influence of novel gene variants in the pathogenesis of polygenic obesity. Therefore, the aims of the present study are to validate the obesity-associations with the GWA identified *INSIG2* rs7566605 and *PFKP* rs6602024 variants, through case-control studies and analyses of obesity-related quantitative traits. Further, since gene-environment and gene-gene interactions may modulate the effect the variants exert on BMI, analyses are extended to investigate such interactions, focusing on physical activity as an environmental risk factor and on GWA identified obesity variants, reaching a stringent genome-wide significance threshold, (*FTO* rs9939609 and *MC4R* rs17782313) as genetic risk factors.

## Materials and Methods

### Study samples

The *INSIG2* rs7566605 and *PFKP* rs6602024 variants were genotyped in 18,014 subjects ascertained from four different study groups; 1) the Inter99 cohort, which is a population-based, randomized, non-pharmacological intervention study of middle-aged subjects for the prevention of ischemic heart disease (*n* = 6,514), conducted at the Research Centre for Prevention and Health in Glostrup, Copenhagen (ClinicalTrials.gov ID-no: NCT00289237) [33]; 2) the ADDITION screening cohort, Denmark (Anglo–Danish–Dutch Study of Intensive Treatment in People with Screen-Detected Diabetes in Primary Care) (ClinicalTrials.gov ID-no: NCT00237548) [34], which is a population-based, high-risk screening and intervention study for type 2 diabetes in general practice (*n* = 8,662); 3) a population-based group of unrelated middle-aged subjects (*n* = 680) examined at Steno Diabetes Center; and 4) unrelated type 2 diabetic patients (*n* = 2,158) sampled through the out-patient clinic at Steno Diabetes Center. The combined study sample refers to the combination of these four study groups. In the combined study sample 1,914 had screen-detected and 2,302 had known type 2 diabetes, 5,512 were normal weight, 7,458 were overweight and 5,044 were obese. All participants in study group 1 and 3 underwent a standard 75 g oral glucose tolerance test. Type 2 diabetes was defined according to the World Health Organization [35]. Overweight and obesity were defined as 25≤BMI<30 and BMI≥30 kg/m^2^, respectively. All study participants were Danes by self-report. Informed written consent was obtained from all subjects before participation. The studies were approved by the regional Ethics Committees (ethics committee, Copenhagen County for study group 1,3 and 4 and ethics committee, Aarhus County for study group 2) and were in accordance with the principles of the *Helsinki Declaration*.

### Biochemical and anthropometrical measurements

In all four study groups body weight and height were measured in light indoor clothes and without shoes. BMI was defined as weight in kilograms divided by height in meters squared (kg/m^2^). Waist circumference (cm) was measured with subjects in standing position midway between the iliac crest and the lower costal margin. In study group 1, 3 and 4 blood samples were drawn after a 12-hour overnight fast and serum triglycerides, total cholesterol and high density lipoprotein (HDL) -cholesterol were determined using enzymatic colourimetric methods (GPO-PAP and CHOD-PAP; Roche Molecular Biochemicals, Mannheim, Germany). Serum insulin levels excluding des(31,32)- and intact proinsulin were measured using the AutoDELFIA insulin kit (Perkin-Elmer, Wallac, Turku, Finland). Plasma glucose was analysed using a glucose oxidase method (Granutest; Merck, Darmstadt, Germany) [36]. The level of physical activity in study group 1 was self-reported by questionnaire and divided into five categories as; physically passive and light, medium, hard or very hard physically active [36]. These categories were combined in two ways in the analyses; three groups defined as physically passive, light or medium physically active, hard or very hard physically active, and in two groups defined as physically passive and physically active including all four levels of physical activity.

### Genotyping

The *INSIG2* rs7566605 and *PFKP* rs6602024 variants were genotyped in the combined study sample using Taqman allelic discrimination (KBioscience, Herts, UK). The discordances between 686 random duplicate samples were 0.3% and 0.0% respectively. Genotyping success rates were 96.8% for both variants, and the minor allele frequencies (MAF) were 32.9% and 10.8%, respectively. The *FTO* rs9939609 and *MC4R* rs17782313 variants used in the gene-gene interaction analyses were genotyped using the same technique. The discordances between 1,464 and 721 random duplicate samples were 0.3% and 0.0% respectively. Genotyping success rates were 97.4% and 95.8%, and the MAF were 41.5% and 25.2%, respectively. All genotype groups obeyed Hardy-Weinberg equilibrium.

### Statistical analyses

In case-control studies of overweight and obesity, logistic regression was applied to examine differences in genotype distributions between affected and unaffected subjects, applying a recessive model for the *INSIG2* rs7566605 variant, and an additive model for the *PFKP* rs6602024 variant. Adjustments for age, sex and study group were introduced analysing the combined study sample, and for age and sex when analysing the population-based Inter99 study sample. A general linear model was used to test quantitative traits for differences between genotype groups applying the prior stated genetic model for each variant. Adjustment for sex, age, study group and BMI was applied when appropriate. Linear models extended with environmental parameters were used to test for gene-environment interactions using an analysis of variance (ANOVA) test, treating physical activity as a categorical variable. Likewise, additional genetic parameters were added to a linear model, when analysing gene-gene interactions with GWA identified obesity variants. All analyses were performed in RGui version 2.6.2 [37]. *p*-values<0.05 were considered significant. A test for homogeneity between the Inter99 cohort, the ADDITION cohort, the population-based and type 2 diabetic patient group from Steno Diabetes Center, was performed by means of the Mantel-Haenszel method (fixed effects model), revealing no significant heterogeneity between the study groups (*p* = 0.5). Statistical power was determined using the CaTS power calculator version 0.0.2. To get robust *p*-values for the gene-environment and gene-gene interactions, we used a permutation procedure where the phenotype labels where randomly permuted between individuals. The lowest *p*-value from all iterations (*n* = 1,000) were saved, and used as an empirical distribution to obtain robust *p*-values which are corrected for multiple testing.

## Results

Using the population-based Inter99 cohort as reference, we found that the prevalence of overweight and obesity was 39% and 17%, respectively, in the Danish population. This gives us a statistical power of 100% observing association between a variant with a MAF of 10% and both overweight and obesity with a relative risk of 1.2, applying an additive model. For a variant with a MAF of 30%, we have a statistical power of 97% and 72% observing an association between overweight and obesity respectively with a relative risk of 1.2, when applying a recessive model.

Potential associations of the *INSIG2* rs7566605 C-allele and the *PFKP* rs6602024 A-allele with overweight and obesity were evaluated by performing case-control studies in the combined study sample and further in the population-based Inter99 cohort, to elucidate the effect on a population-based level, however, no association was observed (Table 1).

**Table 1:** Case-control study of the *INSIG2* rs7566605 and *PFKP* rs6602024 variants in relation to overweight and obesity.

*INSIG2* rs7566605	*n* (men/women)	Genotype distribution *n* GG/GC/CC (%)	MAF (95% CI)	*p*_rec_	OR_rec_ (95% CI)
Combined study sample* ** (** ***n*** ** = 16,781)**					
Controls	5,106 (2,113/2,993)	2,264/2,274/568 (44/45/11)	33.4 (32.5–34.3)		
Overweight cases	6,973 (4,332/2,641)	3,162/3,056/755 (45/44/11)	32.7 (32.0–33.5)	0.7	0.98 (0.87–1.10)
Obese cases	4,702 (2,425/2,277)	2,118/2,077/507 (45/44/11)	32.9 (31.9–33.8)	0.8	1.02 (0.89–1.16)
***Population-based Inter99 cohort*** ** ( ***n*** ** = 6,158)**					
Controls	2,708 (1,038/1,670)	1,176/1,225/307 (44/45/11)	34.0 (32.7–35.2)		
Overweight cases	2,391 (1,449/942)	1,079/1,060/252 (45/44/11)	32.7 (31.4–34.1)	0.4	0.92 (0.77–1.10)
Obese cases	1,059 (524/535)	472/454/133 (44/43/13)	34.0 (32.0–36.1)	0.2	1.15 (0.93–1.44)

*PFKP* rs6602024	n (men/women)	Genotype distribution *n* GG/GA/AA (%)	MAF (95% CI)	*p*_add_	OR_add_
Combined study sample* ** (** ***n*** ** = 16,741)**					
Controls	5,095 (2,110/2,985)	4,054/980/61 (80/19/1)	10.8 (10.2–11.4)		
Overweight cases	6,971 (4,334/2,637)	5,599/1,302/70 (80/19/1)	10.3 (9.8–10.9)	0.1	0.93 (0.85–1.01)
Obese cases	4,675 (2,399/2,276)	3,680/947/48 (79/20/1)	11.2 (10.5–11.8)	0.7	1.02 (0.92–1.12)
***Population-based Inter99 cohort*** ** ( ***n*** ** = 6,144)**					
Controls	2,700 (1,035/1,665)	2,176/493/31 (81/18/1)	10.3 (9.5–11.1)		
Overweight cases	2,386 (1,443/943)	1,949/413/24 (82/17/1)	9.7 (8.8–10.5)	0.2	0.92 (0.81–1.05)
Obese cases	1,058 (522/536)	835/213/10 (79/20/1)	11.0 (9.7–12.4)	0.4	1.07 (0.91–1.26)

Data are number of subjects, divided into genotype groups (% in each group), frequencies of the minor allele (MAF) in percentages (95% CI) and odds ratio (OR) for the applied genetic model (95% CI). Differences in genotype distribution was evaluated using logistic regression, applying a recessive model (*p*_rec_) for the *INSIG2* rs7566605 variant (GG/GC vs. CC) and an additive model (*p*_add_) for the *PFKP* rs6602024 variant (GG vs. GA vs. AA). ^*^In the combined study sample (where all four study groups, the Inter99 cohort, the ADDITION cohort, the SDC population-based and type 2 diabetes sample were included) *p*-values were adjusted for age, sex, and study group, whereas *p*-values in the ^**^population-based Inter99 cohort were adjusted for age and sex. Controls were defined as BMI<25, overweight cases as 25≤BMI<30 and obese cases as BMI≥30 kg/m^2^ respectively.

The two variants were furthermore investigated for influence on quantitative obesity-related traits in the combined study sample and in the population-based Inter99 cohort excluding known type 2 diabetic patients. No association of obesity risk alleles with BMI, body weight or waist circumference could be shown (Table 2).

**Table 2:** Quantitative obesity-related traits in the combined study sample and the population-based Inter99 cohort.

	*INSIG2* rs7566605				*PFKP* rs6602024			
	GG	GC	CC	*p*_rec_	GG	GA	AA	*p*_add_
Combined study sample*								
***n*** ** (men/women)**	6,658 (3,482/3,176)	6,552 (3,467/3,085)	1,619 (836/783)		11,806 (6,177/5,629)	2,840 (1,513/1,327)	150 (71/79)	
**Age (years)**	55±10	55±10	54±10		54±10	55±10	55±10	
**BMI (kg/m^2^)**	27.6±4.8	27.5±4.9	27.5±5.0	0.9	27.5±4.9	27.6±4.9	27.6±5.3	0.8
**Body weight (kg)**	81.1±16.2	81.1±16.2	80.9±16.7	0.9	81.0±16.2	81.3±16.6	79.4±15.3	1
**Waist (cm)**	92.7±14.4	92.5±14.1	92.3±14.6	0.9	92.5±14.2	93.0±14.4	91.7±15.5	0.6
Population-based Inter99 cohort**								
***n*** ** (men/women)**	2,559 (1,271/1,288)	2,559 (1,282/1,277)	652 (317/335)		4,642 (2,303/2,339)	1,056 (532/524)	60 (25/35)	
**Age (years)**	46±8	46±8	46±8		46±8	46±8	46±8	
**BMI (kg/m^2^)**	26.2±4.5	26.1±4.5	26.4±4.5	0.2	26.2±4.5	26.3±4.7	25.9±4.2	0.9
**Body weight (kg)**	78.0±15.7	78.0±15.9	78.3±17.2	0.4	78.1±15.9	78.3±16.4	75.1±13.4	0.9
**Waist (cm)**	86.5±13.2	86.4±13.1	86.7±13.9	0.4	86.4±13.2	87.0±13.3	84.4±12.4	0.6

Data are means±standard deviation. *p*-values were calculated assuming a recessive model (*p*_rec_) for *INSIG2* rs7566605 variant (GG/GC vs. CC) and an additive model (*p*_add_) for *PFKP* rs6602024 variant (GG vs. GA vs. AA) in the combined study sample (where all four study groups, the Inter99 cohort, the ADDITION cohort, the SDC population-based and type 2 diabetes sample were included) and the population-based Inter99 cohort. Known type 2 diabetic patients were excluded from the analyses. ^*^Adjustments for the effect of age, sex and study group was introduced for the combined study sample, and for age and sex in the ^**^population-based Inter99 cohort.

Since the *INSIG2* rs7566605 C-allele has been proposed to exert a larger effect in individuals already predisposed to obesity, we investigated the effect of the variant in the obese subgroup of the combined study sample (*n* = 3,878), with a mean BMI of 32.8 kg/m^2^, however, no genotype effect was found (data not shown).

In the population-based Inter99 cohort, serum insulin and plasma glucose measures during an oral glucose tolerance test were available, together with fasting levels of serum triglyceride and HDL-cholesterol. However, none of these measures were associated with *INSIG2* rs7566605 or *PFKP* rs6602024 genotypes (data not shown).

**Figure 1:**
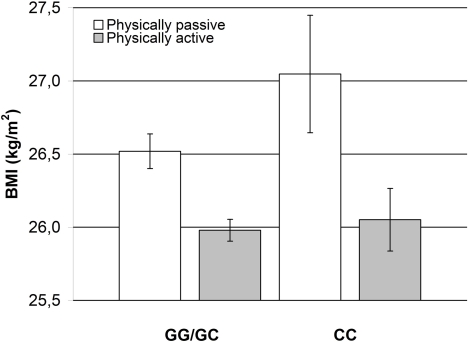
Effect of physical activity on the impact of the *INSIG2* rs7566605 genotype on BMI. Participants (*n* = 5,604) from the population-based Inter99 cohort, were divided according to self-reported physical activity categorised as physically passive and physically active and stratified according to *INSIG2* rs7566605 genotype applying a recessive model. Bars indicate mean BMI, and error bars indicate standard error. The number of participants (physically passive/physically active) are (1,712/3,259) for G-allele carriers and (202/431) for homozygous C-allele carriers. We tested for interaction effects using linear models, with or without, interaction parameters for physical activity compared by an ANOVA test (*p*_Int_ = 0.004).

In analyses of gene-environment interactions comprising a total of 5,604 individuals from the population-based Inter99 cohort, excluding known type 2 diabetes patients, we found an interaction between *INSIG2* rs7566605 genotype and the level of self-reported physical activity. When dividing subjects into three groups categorised as physically passive, light or medium physically active and hard or very hard physically active, a difference in BMI level was only observed between the group of physically passive homozygous C-allele carriers and physically passive G-allele carriers (*p*_Int_ = 0.004). No difference in BMI was observed between genotype groups for light or medium physically active or hard or very hard physically active. Therefore, we tested physically passive against physically active (light to very hard physically active) (*p*_Int_ = 0.004), and found that physically passive homozygous C-allele carriers had a BMI 1.00 (SE 0.46) kg/m^2^ higher than physically active homozygous C-allele carriers whereas physically passive G-allele carriers only had a BMI 0.54 (SE 0.14) kg/m^2^ higher than physically active G-allele carriers (Figure 1). This is also reflected in a difference of 0.53 (SE 0.42) kg/m^2^ between physically passive homozygous C-allele carriers and physically passive G-allele carriers. The robustness of the *p*-value was verified by the empirical value obtained by the permutation procedure (*p* = 0.005). No interaction between the *PFKP* rs6602024 variant and physical activity was demonstrated.

We further investigated potential interactions between the rs7566605 and rs66020245 variants and GWA identified obesity variants reaching the stringent significance threshold; rs9939609 located in *FTO* and rs17782313 located downstream from *MC4R*, however, no interactions were observed (data not shown).

## Discussion

Analysing 18,014 Danish individuals, we failed to demonstrate a correlation between the *INSIG2* rs7566605 C-allele and the *PFKP* rs6602024 A-allele and excessive body fat accumulation both in case-control studies of overweight and obesity, and when analysing quantitative obesity-related traits. The obesity-association of *PFKP* rs6602024 was not successfully replicated in the initial GWA study, which was suggested to be due to the relatively low risk allele frequency of the variant resulting in only a small fraction of the population being affected. However, the variant exerted a relatively large effect size on BMI in the replication cohorts, with homozygous A-allele carriers having a BMI ∼3 units higher than G-allele carriers, and therefore it was proposed that larger population samples might be needed in order to reach statistical significance [17]. We did, however, not replicate the association between *PFKP* rs6602024 and obesity measures, either using an additive or recessive model. On the contrary we observed slightly lower BMI, body weight and waist circumference among homozygous *PFKP* rs6602024 A-allele carriers. The failure to replicate the association between *PFKP* rs6602024 and different measures of obesity in the present study could be due to differences in linkage disequilibrium patterns between the population in which the variant was first identified [17] and our study material. An independent GWA study in unrelated U.S. Caucasians has reported an association between three other polymorphisms in *PFKP* and excessive body fat accumulation [2]. Hence, *PFKP* could in theory be a true obesity susceptibility gene, with rs6602024 failing to be a marker for the functional variant in our population.

Several studies have failed to validate the initial GWA finding of the *INSIG2* rs7566605 C-allele contributing to the pathogenesis of obesity. Despite the large-scale study samples included in our study, we were not able to confirm the proposed association, in neither case-control nor quantitative settings. The effect of the *INSIG2* rs7566605 variant on BMI has been proposed to be predominant in already obese subjects, but neither in an obese subgroup did we observe an association. However, the effect of some genetic variants is only exerted in combination with environmental or other genetic risk factors [38]. When taking the level of physical activity into account, we detected an *INSIG2* rs7566605 genotype effect on the level of BMI. Physical inactivity results in an increased BMI level in both G-allele carriers and homozygous C-allele carriers. In the group of G-allele carriers the effect of physical inactivity on BMI level is 0.54 (SE 0.14) kg/m^2^, whereas the effect is more pronounced in homozygous C-allele carriers increasing the BMI level by 1.00 (SE 0.46) kg/m^2^. This indicates an interaction between the *INSIG2* rs7566605 variant and physical activity. This is supported by the observation that the BMI level in the subgroup of physically passive is 0.53 (SE 0.42) kg/m^2^ higher in homozygous C-allele carries compared with physically passive G-allele carriers. However, this interaction analysis is merely explorative, since it is based on self-reported measures, and replication in statistically well-powered populations with more precise physiological physical activity measures is imperative.

We can thus conclude that common variation in *INSIG2* and *PFKP*, both candidate genes arising from GWA studies, do not play a significant role in the pathogenesis of obesity in the Danish population. Still, if replicated common variation in *INSIG2* might, contribute to common forms obesity through interaction with a low level of physical activity.
